# The effect of anti-IL-6 receptor antibody for the treatment of McH-lpr/lpr-RA1 mice that spontaneously developed destructive arthritis and enthesitis

**DOI:** 10.1186/s12891-019-2664-3

**Published:** 2019-06-15

**Authors:** Takuya Izumiyama, Yu Mori, Shiro Mori, Naoko Mori, Tetsuya Kodama, Eiji Itoi

**Affiliations:** 1 Department of Orthopaedic Surgery, Tohoku University Graduate School of Medicine, 1-1, Seiryo-machi, Aoba-ku, Sendai, Miyagi 980-8574 Japan; 2 Laboratory of Biomedical Engineering for Cancer, Department of Biomedical Engineering, Graduate School of Biomedical Engineering, Tohoku University, 4-1, Seiryo-machi, Aoba-ku, Sendai, Miyagi 980-8575 Japan; 3 Department of Diagnostic Radiology, Tohoku University Graduate School of Medicine, 1-1, Seiryo-machi, Aoba-ku, Sendai, Miyagi 980-8574 Japan

**Keywords:** Arthritis, Enthesitis, Anti-IL-6 receptor antibody, IVIS

## Abstract

**Background:**

McH-lpr/lpr-RA1 mice are a new strain of mice which spontaneously develop destructive arthritis and enthesitis in the ankle. There is no published data that drug treatment has been trialed on these mice. This study examined the effect of the mouse anti-IL-6 receptor antibody, MR16–1, for the treatment of arthritis and enthesitis in McH-lpr/lpr-RA1 mice.

**Methods:**

Male McH-lpr/lpr-RA1 mice were randomly divided into control and treatment groups. MR16–1 was administered from 10 weeks of age for the treatment group. Saline was applied for the control group. The drug was administered once a week, at an initial dose of 2 mg, then maintained at 0.5 mg once per week thereafter. The effects were evaluated by the histopathological synovitis score, in vivo imaging using indocyanine green liposomes, and analysis of the gene expression of inflammatory cytokines.

**Results:**

Tissue analyses were carried out at 14, 17 and 20 weeks of age. The synovitis scores of treated groups were significantly lower compared with those of the control group at 14 and 17 weeks of age. The kappa coefficient was 0.77. However, progression of entheseal ossification persisted in the MR16–1 treated group. In vivo imaging using indocyanine green liposomes showed significant decreases in signal intensities of treated groups at week 14, but no significant differences were observed at week 18. Blood serum amyloid A levels in treated groups were significantly lower at 17 weeks of age. The gene expression levels of *Tnf* and *Il17* were also significantly lower in MR16–1 treated groups.

**Conclusions:**

Administration of the anti-IL-6 receptor antibody is effective for the treatment of synovitis and bone destruction of McH-lpr/lpr-RA1 mice. McH-lpr/lpr-RA1 mice may be a suitable experimental model for the development of new treatments for destructive arthritis and enthesitis. IL-6 signal blockade could contribute to the treatment of destructive arthritis, and further studies should be carried out to confirm its potential in the prevention of enthesopathy developed to ossification.

## Background

Autoimmune arthritis diseases with entheseal inflammation show complex pathological features, including synovitis, bone erosion, enthesitis [1]. The imbalance of bone and cartilage formation and the destruction of joint structure results in the structural changes observed in these diseases. Rheumatoid arthritis is characterized by synovial proliferation and erosion of joint cartilage due to chronic inflammation [2]. On the other hand, autoimmune arthritis with entheseal inflammation like as psoriatic arthrits involves a distinct remodeling process leading to entheseal ossification with synovial proliferation and bone erosion [3, 4]. The histological characteristics of entheseal ossification include the proliferation of cartilage formation, and subsequent replacement of cartilage by bone (endochondral ossification) [5, 6]. Recent studies have reported that IL-17 and IL-22 signaling molecules released from IL-23 positive T cells are involved in entheseal chondral proliferation and ossification [7, 8]. Patients with ossifying enthesitis are typically treated with tumor necrosis factor (TNF)-α inhibitors. However, the prevention of entheseal ossification is recognized as being difficult especially in advanced stages of the disease [9–11]. Moreover, animal models of synovitis with concomitant entheseal ossification are yet to be established. It is necessary to study animal models of synovitis with intercurrent entheseal ossification to elucidate the pathological mechanisms of the disease, and develop new treatment methods.

Our research group have previously reported the development of a murine model with spontaneous, progressive synovitis and enthesitis in ankle joints [12]. Backcross generation mice were prepared using a non-arthritis strain of mice, C3H/HeJ-lpr/lpr (C3H/lpr), MRL/lpr × (MRL/lpr × C3H/lpr) F1. Among these N2 mice, we observed development of arthritis of the ankle joints, with macroscopic swelling. We then began to intercross the N2 mice, by selection based on swelling of the ankle joints. Finally, we established a novel recombinant strain of mice, designated McH-lpr/lpr-RA1, which showed a high incidence of arthritis with enthesopathy. Analysis of the destructive arthritis and enthesitis processes may elucidate the fundamental mechanisms of peripheral inflammation in spondyloarthritis.

The anti-IL-6 receptor monoclonal antibody is available for clinical use in patients with rheumatoid arthritis, and has been shown to have therapeutic effects in the treatment of severe rheumatoid arthritis [13]. The effect of anti-IL-6 treatment in patients with ankylosing spondylitis has also been reported — studies revealed that the anti-IL-6 receptor antibody did not prevent the progression of spinal changes associated with ankylosis [14]. Furthermore, the effect of treatment with anti-IL-6 receptor antibody on peripheral arthritis of spondyloarthritis has also been reported, but the study reported mainly about insufficient effects on spinal disorder and its use in entheseal inflammation and ossification with destructive arthritis remains unclear [15]. MR16–1 is a rat anti-IL-6 receptor monoclonal antibody, as previously described in the literature [16]. MR16–1 is used in many experimental disease models to assess the effects of blocking IL-6 signaling [17–23]. McH-lpr/lpr-RA1 mice may provide a suitable animal model to assess the therapeutic effects of IL-6 signal blockade in entheseal inflammation and ossification, and the administration of MR16–1 will determine the potential treatment effects of IL-6 ligand and receptor blockade.

The purpose of the present study is to investigate the effect of MR16–1 for the treatment of ankle joint arthritis with enthesopathy, and the mechanism of entheseal inflammatory change and ossification in the experimental murine model of McH-lpr/lpr-RA1 mice. We hypothesized that IL-6 signal blockade would suppress the progression of synovial proliferation and entheseal ossification in McH-lpr/lpr-RA1 mice.

## Methods

### Animals

McH/lpr-RA1 mice were generated using F54 C3H/HeJ-*lpr/lpr* and MRL/lpr × (MRL/lpr × C3H/lpr) mice in the animal unit of Tohoku University Medical School. This recombinant congenic strain of mice was designated McH/lpr-RA1 as previously described in the literature [12]. All mice were housed in the animal unit of Tohoku University Medical School, an environmentally controlled and specific pathogen-free facility. Animal protocols were reviewed and approved by the Tohoku University Animal Studies Committee. The animal experiments approval number of our institute was 2015-MdA-247-1. The animals were maintained in individually-ventilated cage (225 × 338 × 140 mm) at 22 ± 2 °C and 40 ± 20% humidity, receiving water and specific animal pellet-type laboratory-animal food. All experiments were performed using week 10 male mice. The mice were randomly allocated to treatment and control groups at week 10. The animals were euthanized in a carbon dioxide gas chamber at 14–20 weeks of age.

### Treatment of mice

IL-6 signal blockade was performed with an intraperitoneal injection of 2 mg of rat anti-mouse IL-6R mAb (MR16–1, a kind gift from Chugai Pharmaceutical, Tokyo, Japan), once in the first treatment (week 10). Thereafter, 0.5 mg of MR 16–1 was administered once a week until 20 weeks of age as previously described in the literature [24] Phosphate buffered saline (PBS) was administered on the same schedule as a negative control.

### Enzyme-linked immunosorbent assay

Serum amyloid A (SAA) and IL-6 levels were determined using an enzyme-linked immunosorbent assay (ELISA) kit for SAA and IL-6 (Biosource, Camarillo, CA and R&D Systems Inc., Minneapolis, MN, USA) according to the manufacturer’s recommendations at 14 and 17 weeks of age (*n* = 5 for each group). Briefly, serum samples were diluted 1:200 in assay diluent and incubated with conjugated anti-mouse SAA antibody. Serum samples were incubated with anti-mouse IL-6 antibody without dilution. Substrate tetramethylbenzidine was added, samples were read at OD450 nm and results were analyzed using the four-parameter fit to determine SAA values.

### Histomorphometric analysis

Ankle joints were harvested at 14, 17 and 20 weeks of age and decalcified by soaking in 220 mM/L EDTA-Na for 3 weeks. Decalcified samples were sectioned and stained with hematoxylin and eosin for histopathological evaluation of synovitis and ankylosis, as previously described in the literature [25]. The calculated synovitis score was the sum of scores for: enlargement of the synovial lining cell layer (0 points: thickness of 1 layer; 1 point: thickness of 2–3 layers; 2 points: thickness of 4–5 layers; 3 points: thickness of more than 5 layers), density of the resident cells (0 points: normal cellularity; 1 point: slightly increased cellularity; 2 points: moderately increased cellularity; 3 points: greatly increased cellularity, pannus formation and rheumatoid like granulomas might occur) and inflammatory infiltrate (0 points: no inflammatory infiltrate; 1 point: few lymphocytes or plasma cells; 2 points: numerous lymphocytes or plasma cells, sometimes forming follicle-like aggregates; 3 points: dense, band-like inflammatory infiltrate, or numerous large, follicle-like aggregates). The values were summarized and interpreted as follows, 0–1:no synovitis; 2–4: low grade synovitis; 5–9: high grade synovitis. The synovitis score was assessed by two researchers and the kappa coefficient was calculated (*n* = 5 for each group).

### Synthesis of indocyanine green liposomes

Indocyanine green (ICG) liposomes were prepared as previously described in the literature [26–28]. Briefly, 1,2-distearoyl-sn-glycero-3-phosphatidylcholine (DSPC; NOF, Tokyo, Japan) and 1,2-distearoyl-sn-glycero-3-phosphoethanolamine-N-[methoxy-(polyethylene glycol)-2000] (DSPE-PEG[2000-OMe]) (DSPE-PEG:NOF, 94:6 mol/mol) were placed in a pear-shaped flask, and chloroform was added until the lipids were completely dissolved. The chloroform was then evaporated under reduced pressure using a rotary evaporator (NVC-2100/N-1000, Eyela, Tokyo, Japan) until a lipid film remained.

Next, 100 μM of ICG (Daiichi Sankyo, Tokyo, Japan) dissolved in 10 mL PBS was added to the thin lipid film to form multi-lamellar liposome vesicles. After repeated freeze-thaw cycles, the size of the liposomes was adjusted to < 100 nm using extrusion equipment (Northern Lipids Inc., Vancouver, BC, Canada) with four filter sizes (100, 200, 400 and 600 nm; Nuclepore Track-Etch Membrane, Whatman plc, Maidstone, UK). For sterilization, liposomes were passed through a 0.45 μm pore size filter (Millex HV filter unit, Durapore polyvinylidene-difluoride [PVDF] membrane, EMD Millipore, Billerica, MA, USA). Unbound ICG was removed using PD-10 columns. The lipid concentration was measured using the Wako Phospholipids C test (Wako Pure Chemical Industries, Osaka, Japan), and adjusted to 1.0 μmol/mL. Diameters of the ICG liposomes were measured using a particle size analyzer (ELSZ-2, Otsuka Electronics, Osaka, Japan).

### In vivo bioluminescence imaging system analysis

Previous reports indicate that Near-infrared fluorescence imaging system with ICG show significant accumulation in inflamed joints with enhanced permeability and retention (EPR), like as the accumulation observed in tumors [29, 30]. Blood vessel insufficiency of inflamed joints in arthritic experimental models increases the leakiness and permeability, which enhances the accumulation of liposomes in arthritic lesion by EPR [31]. For the detection of the arthritic inflammation and the proliferation of dysfunctional blood vessels, In vivo bioluminescence imaging system (IVIS) analysis was performed at 10, 14 and 18 weeks of age in MR16–1 treated and control mice (*n* = 5 for each group). Two hundred μl of ICG liposomes was administered intravenously. The fluorescence intensity of leaked ICG was measured at 0, 5, 30, 60, 120 and 180 min after injection using an IVIS; Xenogen, Alameda, CA, USA), as previously described in the literature [26, 27].

### Quantitative RT-PCR

Synovial tissue specimens from ankle joints were harvested at 10, 14 and 16 weeks of age (*n* = 5 for each group). Total RNA was extracted using QIAzol Lysis Reagent (Qiagen, Hilden, Germany) and an RNeasy Mini Kit (Qiagen, Hilden, Germany). Synthesis of cDNA from total RNA was carried out using RT buffer, RT random primers, dNTP mix, and Multiscribe reverse transcriptase (Applied Biosystems, Foster city, CA, USA). A total of 9 μL cDNA diluted 1:9 was added to 10 μL Taqman Universal Master Mix II with Uracil N-glycosylase (Applied Biosystems, Foster City, CA, USA). Real-time amplification of the genes was performed using 1 μL ready-to-use Taqman Gene Expression Assays (Applied Biosystems) for *Il6*, *Tnf*, *IL17,* and *gapdh* as an endogenous control (assay IDs: Mm00446190_m1, Mm00443260_g1, Mm00439618_m1, and Mm99999915_g1). Relative gene expression data were analyzed using the delta-delta-Ct method with PCR-efficiency correction using StepOne software version 2.2.2 (Applied Biosystems), as previously described in the literature [32].

### Microcomputed tomography analysis

Microcomputed tomography (micro-CT) imaging was performed at 20 weeks of age (*n* = 5 for each group). Harvested tibiae were stored in 70% ethanol at 4 °C, and analyzed using a micro-CT scanner (Scan Xmate-L090; Comscan Techno Co. Ltd., Kanagawa, Japan) operated at a peak voltage of 75 kV and 100 μA. The scanned region included 505 images from the proximal end of the tibia, at a resolution of 10.4 μm per voxel, and an image size of 516 × 506 pixels. Bone volume (BV; mm^2^), total volume (TV; mm^2^), bone volume fraction (BV/TV; %) and trabecular thickness (mm) were evaluated and calculated from the axial slice of the proximal tibia using TRI/3D-BON software (Ratoc System Engineering Co., Tokyo, Japan), as previously described in the literature [33].

### Statistical analysis

Statistical analyses were performed using JMP software version 13.1 (SAS, Cary, NC, USA). All data are expressed as the mean ± standard error (SE). Statistical significance of the differences between values were evaluated using the Student’s t-test, two-way analysis of variance (ANOVA) with Tukey’s multiple comparison test and two-way ANOVA with Dunn’s multiple comparison test. Values of *p* < 0.05 were regarded as statistically significant. Kappa coefficients were calculated using SPSS version 21 (IBM, Armonk, NY, USA).

## Results

### ELISA assay

**Fig. 1:**
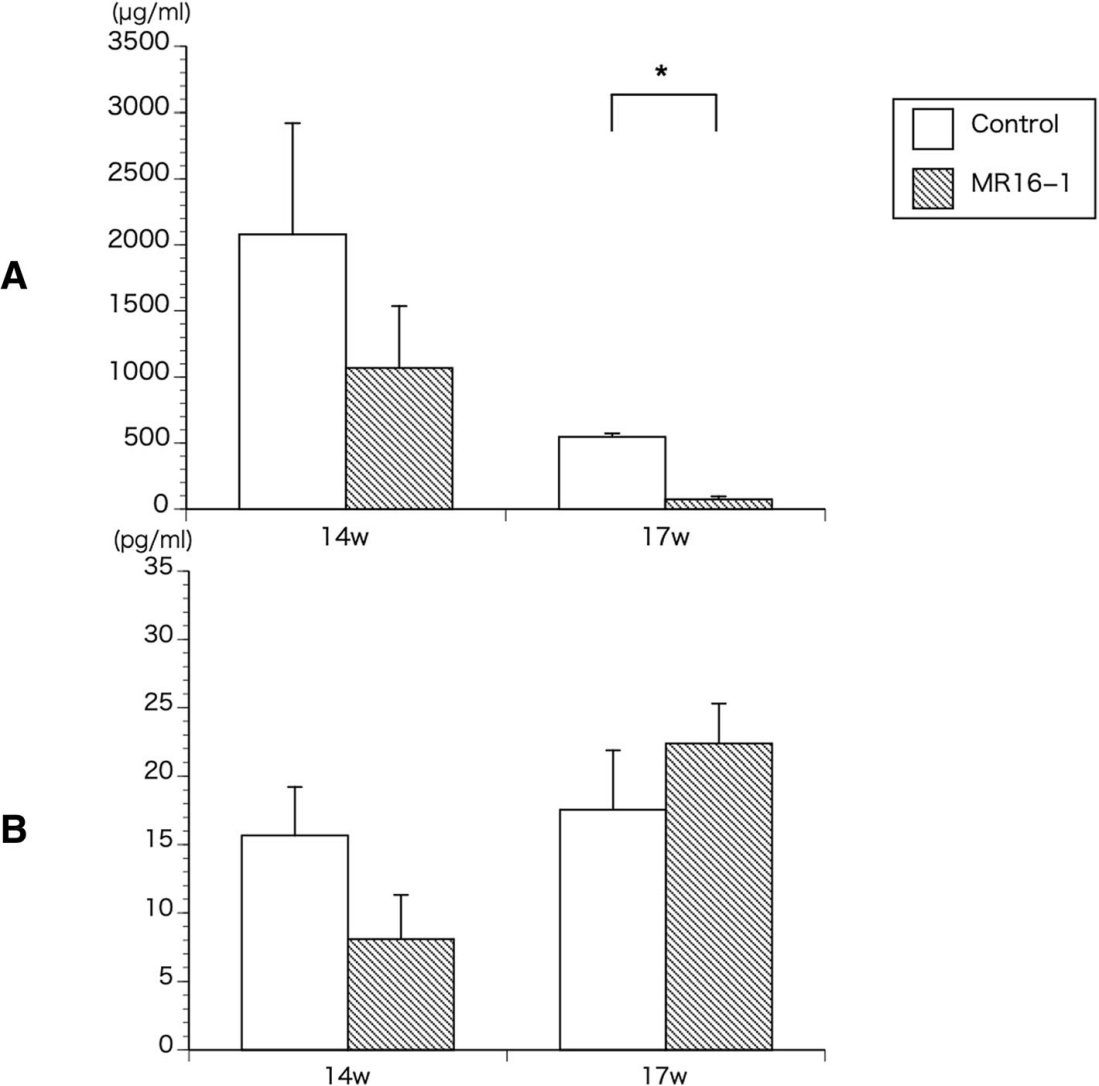
ELISA assays for SAA and IL-6. **a** ELISA assay for SAA at week 14 and 17. Serum SAA level is significantly lower in MR16–1 treated group at week 17. **b** ELISA assay for IL-6 at week 14 and 17. There is no significant difference in serum IL-6 level. Results are expressed as the mean ± standard error (*n* = 5 mice per group). Representative data are shown from three independent experiments with similar results. *p < 0.05 by two-way ANOVA with Dunn’s multiple comparison test

The results of the ELISA assays showed reduced levels of SAA in the MR16–1 treatment group at 14 weeks of age, with significantly decreased levels at week 17 (*p* = 0.04) (Fig. 1a). The results of the assay for serum IL-6 indicated no significant difference between the control and MR16–1 treatment groups at 14 and 17 weeks of age (Fig. 1b).

### Histomorphometric analysis

**Fig. 2:**
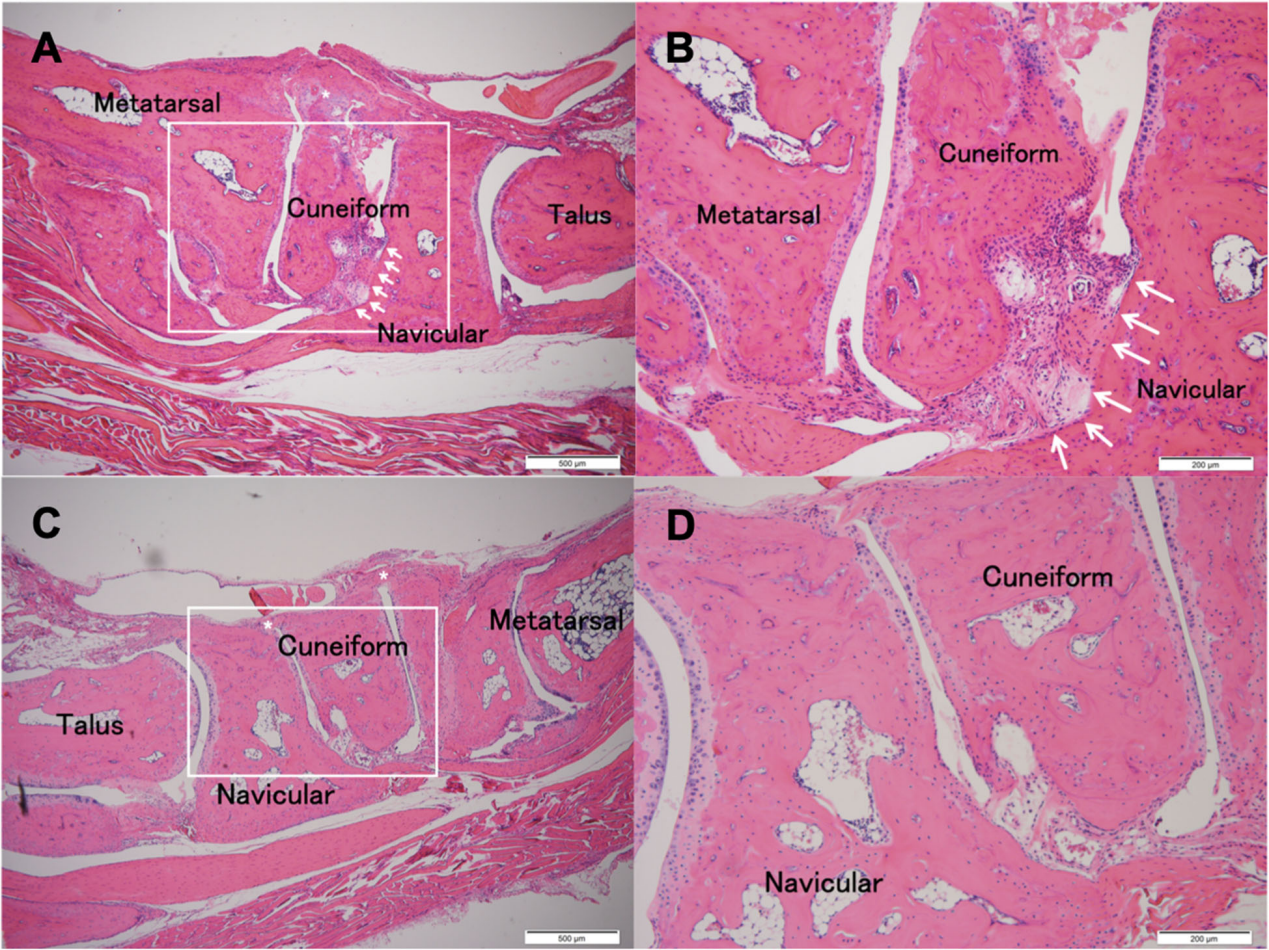
Histological images of control and MR16–1 treated groups at week 14. Representative histological images of ankle joint and foot in control group. **a** is × 40 lower magnification image. **b** is × 100 higher magnification image. Rectangle shows the higher magnification area. Arrows indicate synovial proliferation. * indicates entheseal fibrous and cartilaginous ankylosis. Representative histological images of ankle joint and foot in MR16–1 treatment group. **c** is × 40 lower magnification image. **d** is × 100 higher magnification image. Rectangle shows the higher magnification area. Arrows indicate synovial proliferation. * indicates fibrous and cartilaginous ankylosis

**Fig. 3:**
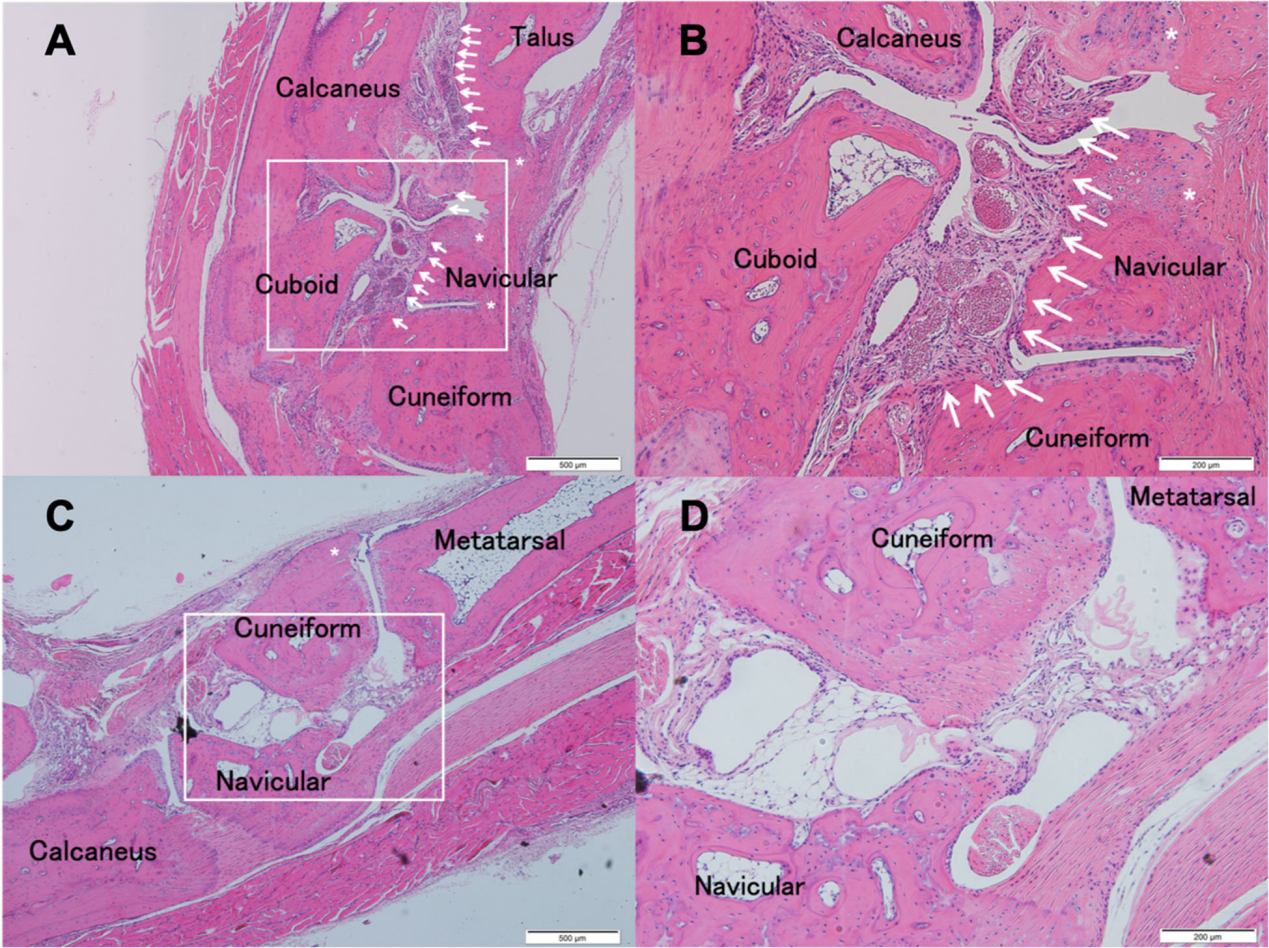
Histological images of control and MR16–1 treated groups at week 17. Representative histological images of ankle joint and foot in control group. **a** is × 40 lower magnification image. **b** is × 100 higher magnification image. Rectangle shows the higher magnification area. Arrows indicate synovial proliferation. * indicates entheseal fibrous and cartilaginous ankylosis. Representative histological images of ankle joint and foot in MR16–1 treatment group. **c** is × 40 lower magnification image. **d** is × 100 higher magnification image. Rectangle shows the higher magnification area. Arrows indicate synovial proliferation. * indicates entheseal fibrous and cartilaginous ankylosis

**Fig. 4:**
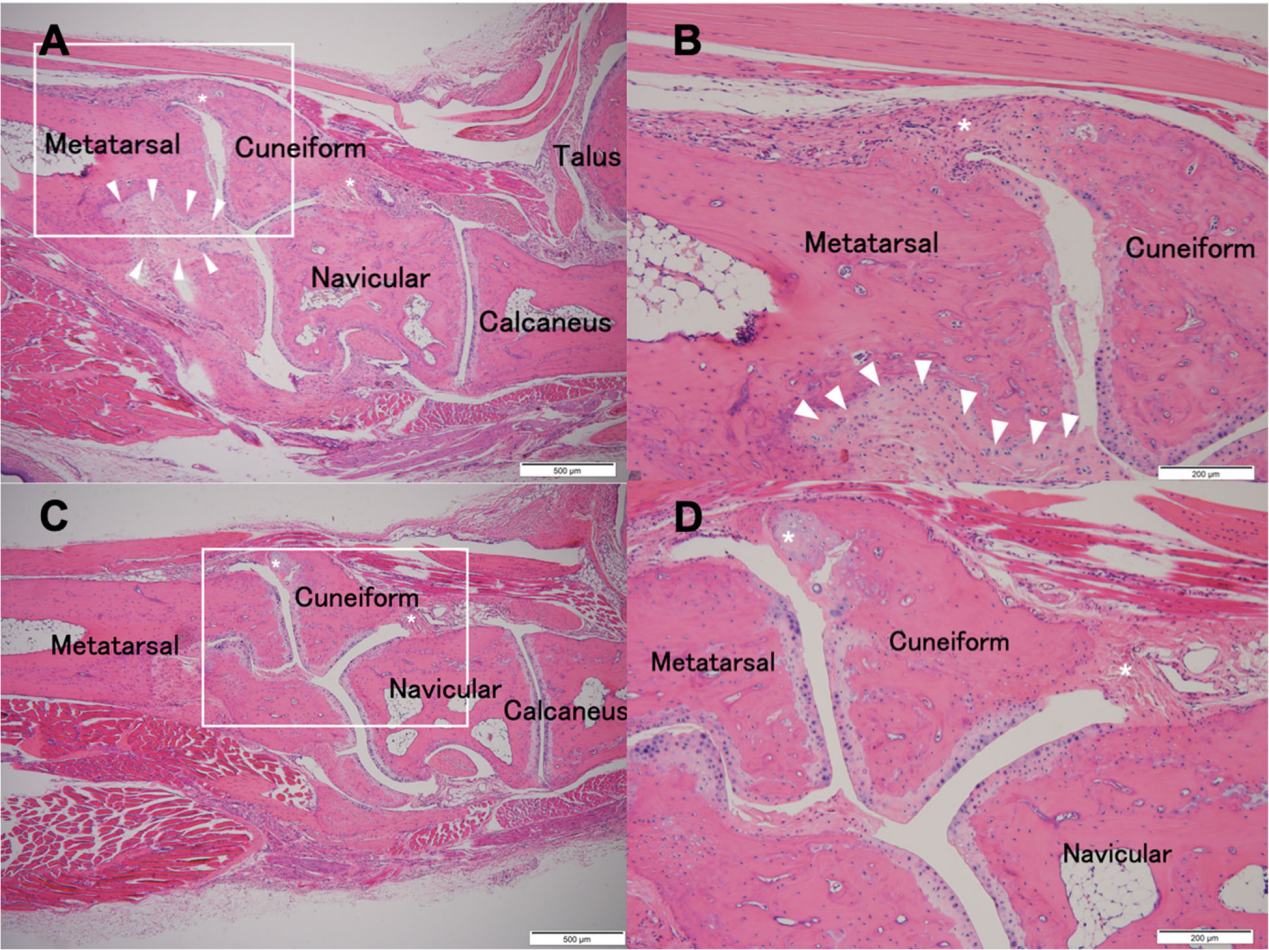
Histological images of control and MR16–1 treated groups at week 20. Representative histological images of ankle joint and foot in control group. **a** is × 40 lower magnification image. **b** is × 100 higher magnification image. Rectangle shows the higher magnification area. Arrow heads indicate subsequent interosseous ankylosis after synovial proliferation. * indicates entheseal fibrous and cartilaginous ankylosis. Representative histological images of ankle joint and foot in MR16–1 treatment group. **c** is × 40 lower magnification image. **d** is × 100 higher magnification image. Rectangle shows the higher magnification area. * indicates entheseal fibrous and cartilaginous ankylosis

**Fig. 5:**
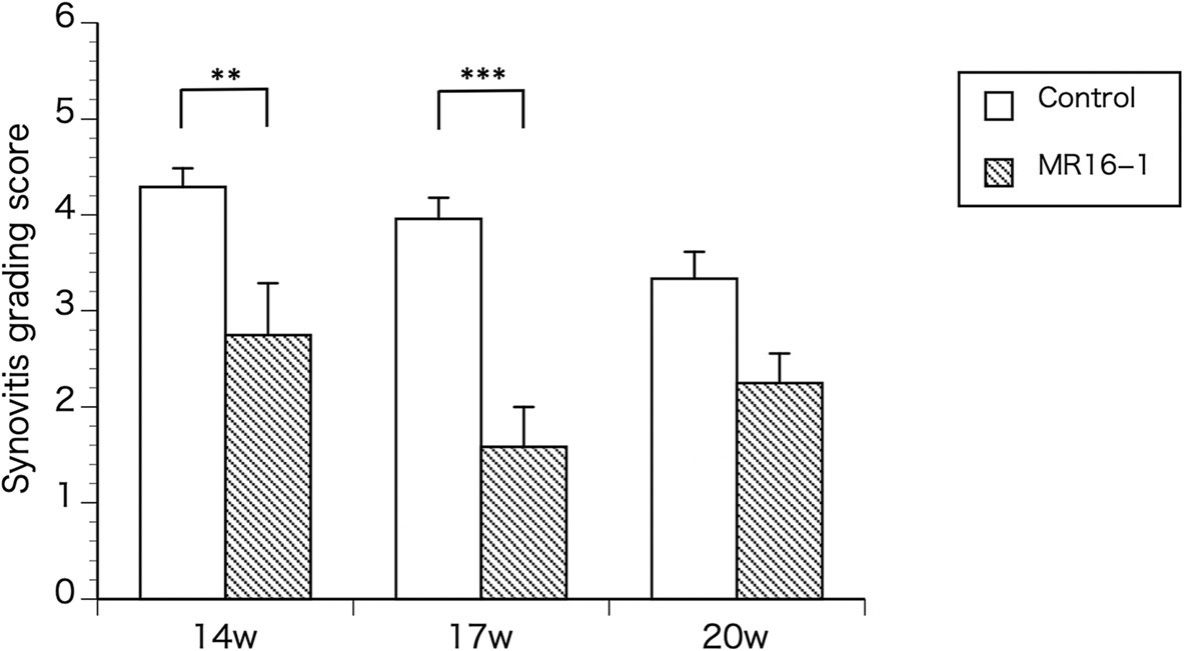
Histomorphometric analyses with the synovitis grading score of foot and ankle joints in control and MR16–1 treated mice. The synovitis grading scores of MR16–1 treatment group are significantly lower in week 14, 17 and 20 compared with those of control group. Results are expressed as the mean ± standard error (*n* = 5 mice per group). Representative data are shown from three independent experiments with similar results. **p < 0.01, ***p < 0.001 by two-way ANOVA with Tukey’s multiple comparison test

At 14 weeks of age, control group mice showed apparent synovitis, pannus formation, entheseal inflammation and ankylosis in the ankle region (Fig. 2a and b). In MR16–1 treated mice, synovitis and pannus formation was suppressed compared with the control group (Fig. 2c and d). At 17 weeks of age, control group mice exhibited acceleration of synovitis, pannus formation and ankle joint ankylosis (Fig. 3a and b). In the MR16–1 treatment group, no synovitis or pannus formation were detected, however, the progression of entheseal inflammatory cells filtration and ankylosis were seen (Fig. 3c and d). At 20 weeks of age, control group mice showed degenerative synovial lesions, advanced pannus formation and joint ankylosing change (Fig. 4a and b). In the MR16–1 treatment group, inflammatory cells filtration and ankylosis of the entheseal area were found, although synovitis and pannus formation were absent (Fig. 4c and d). Histopathological assessments of ankle arthritis were performed by calculation of the synovitis score. The Kappa coefficient was calculated to be 0.77, and the reproducibility was good. At week 14, the synovitis score of the control group was 4.29 ± 019, and that of the MR16–1 group was 2.75 ± 0.53. This indicates a significant difference between the two groups (*p* = 0.0098). At week 17, the synovitis score of the control group was 3.95 ± 0.22, and that of the MR16–1 group was 1.64 ± 0.27. This indicates a significant difference between the two groups (*p* < 0.001). At week 20, the synovitis score of the control group was 3.33 ± 0.28, and that of the MR16–1 group was 2.25 ± 0.3. This indicates no significant difference between the groups (Fig. 5). Even in the MR16–1 group, the synovitis score was not completely suppressed due to the presence of inflammatory cells filtration of entheseal area.

### IVIS analysis

**Fig. 6:**
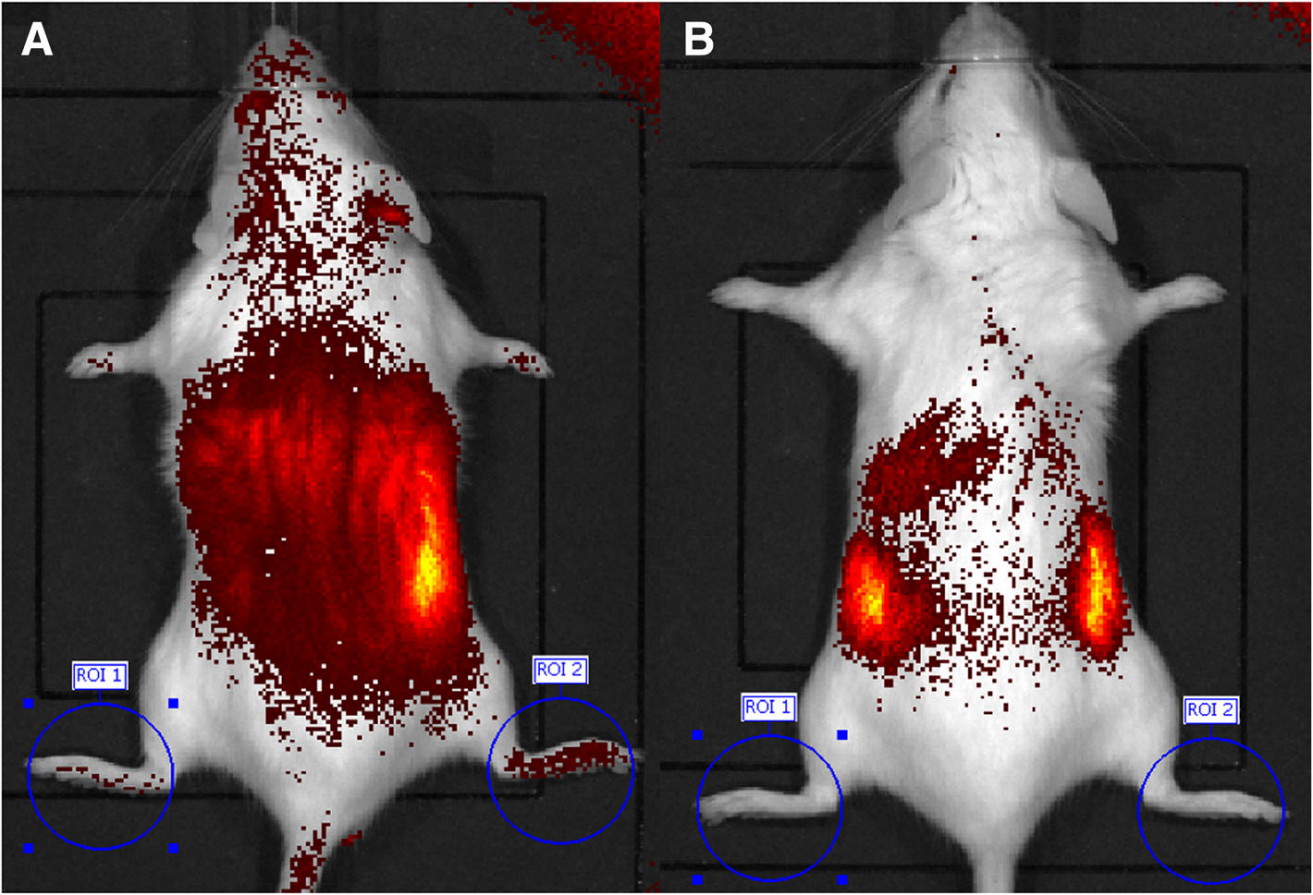
IVIS analyses in control and MR16–1 treated mice. **a** Representative image of IVIS of control mouse at week 14. There are evident signals in both ankles and foots. **b** Representative image of IVIS of MR16–1 treated mouse at week 14. There are no significant uptake signals in ankles and foots

**Fig. 7:**
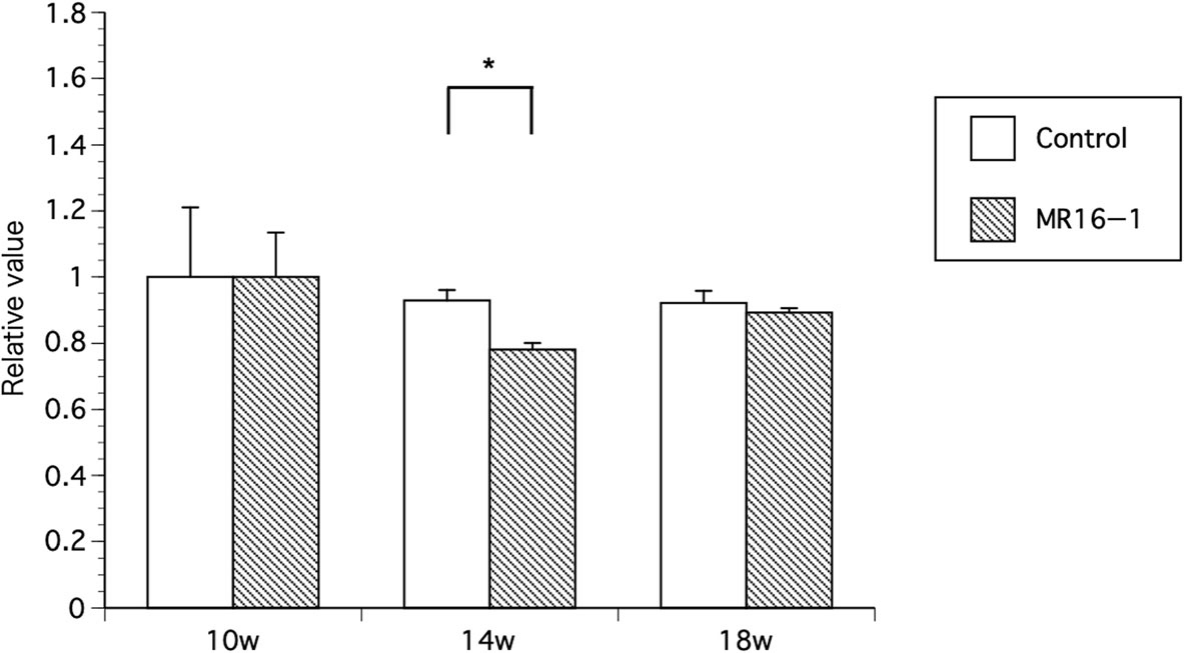
Quantitative analyses of signal intensity of IVIS at week 10, 14, 18. At week 14, the signal intensity of IVIS of ankles and foots is significantly lower in MR16–1 treatment group, compared with the control group. There are no significant changes in week 10 and 18 in both groups. Results are expressed as the mean ± standard error (*n* = 5 mice per group). Representative data are shown from three independent experiments with similar results. * *p* < 0.05 by two-way ANOVA with Dunn’s multiple comparison test

The changes in the signal intensities pre-injection and 120 min after injection were assessed. Representative images of 14 weeks of age control mice and MR16–1 treated mice are shown in Fig. 6a and b. MR16–1 treated mice did not show ICG accumulation in the ankle or foot joints. However, control mice showed obvious accumulation in the foot and ankle joints. The results of IVIS analyses are shown in Fig. 7. Quantitative IVIS was carried out at weeks 10, 14 and 18 in control and MR16–1 treated mice. There was a significant difference in the signal intensities of control and MR16–1 treated mice at week 14 (*p* = 0.041), although there no significant differences were seen at weeks 10 or 18.

### Quantitative RT-PCR

**Fig. 8:**
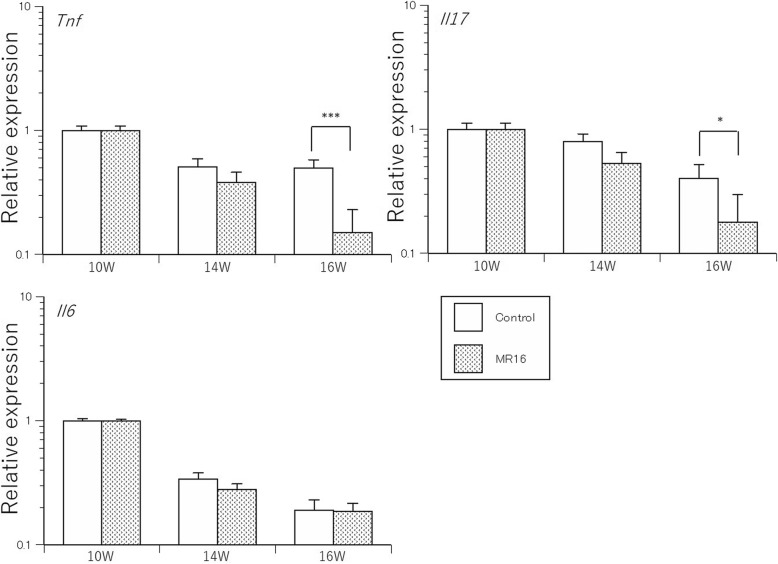
mRNA expression of *Tnf*, *Il17* and *Il6* genes. The expression levels of *Tnf* is significantly lower in MR16–1 treated group at week 16. The expression levels of *Il17* is significantly lower in MR16–1 treated group at week 16. There is no significant difference of *Il6* expression among both groups. Results are expressed as the mean ± standard error (*n* = 5 mice per group). Representative data are shown from three independent experiments with similar results. *p < 0.05, ***p < 0.001 by two-way ANOVA with Tukey’s multiple comparison test

The results of quantitative PCR analyses are shown in Fig. 8. *Tnf* gene expression gradually decreased until week 16 in the MR16–1 treatment group. There was a significant difference in *Tnf* gene expression between the groups at week 16 (*p* = 0.001), which was lower in the MR16–1 treated group than the control group. *Il17* gene expression also gradually decreased until week 16 in the MR16–1 treatment group. There was also a significant difference in *Il17* gene expression between the groups at week 16 (*p* = 0.022), which was lower in MR16–1 treated group. *Il6* gene expression was also evaluated, revealing no significant difference in expression levels between the groups.

### Micro CT analysis

**Fig. 9:**
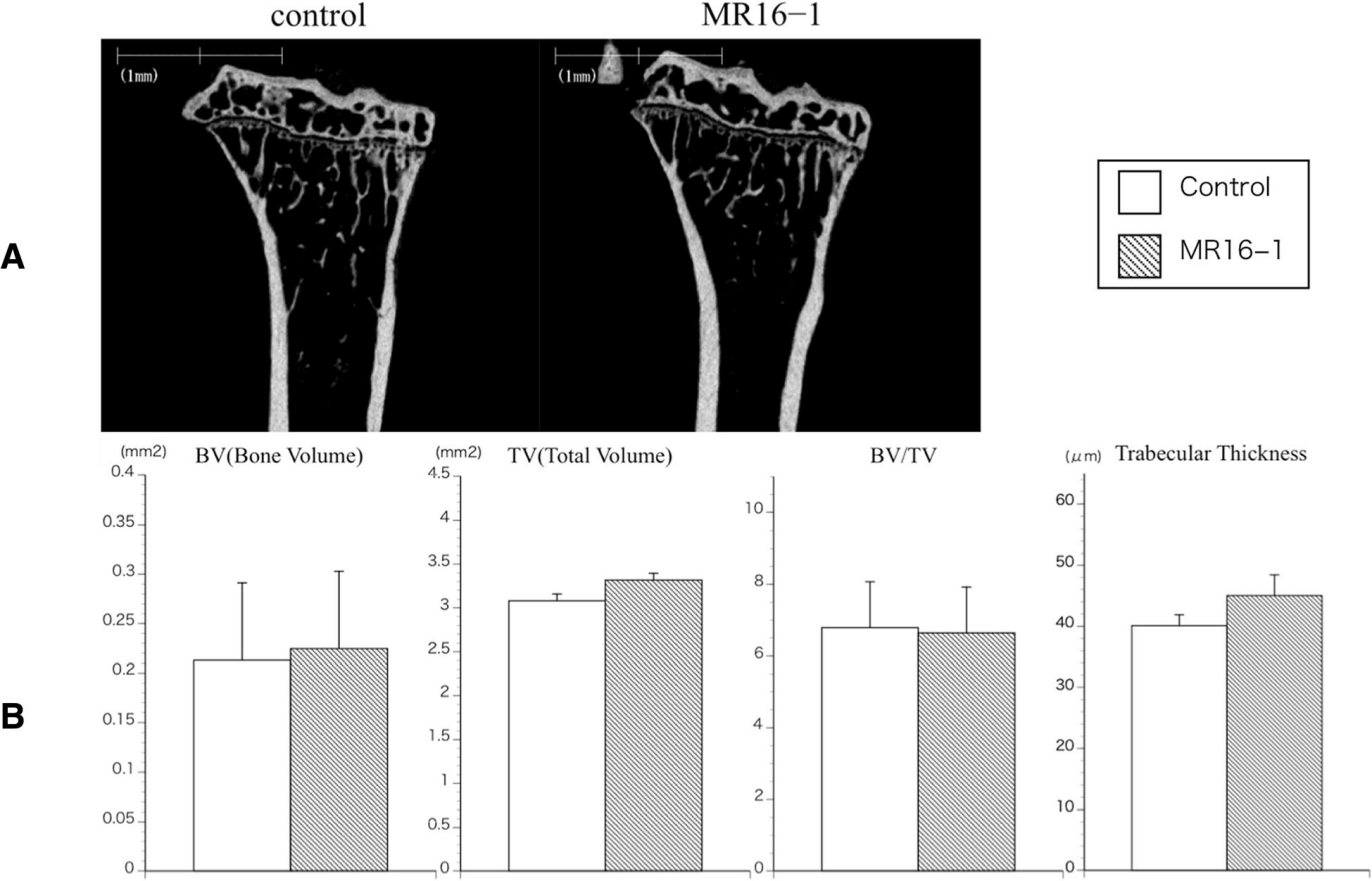
Micro CT image of tibia at week 20 in control and MR16–1 treated groups. **a** Representative coronal reconstruction images of proximal tibia in control and MR16–1 treated groups. **b** Structural parameter analyses of tibia of control and MR16–1 treated mice. Results are expressed as the mean ± standard error (*n* = 5 mice per group). Representative data are shown from two independent experiments with similar results. There are no significant differences in BV, TV, BV/TV and trabecular thickness by Student’s t-test

In the coronal-reconstructed micro CT images at 20 weeks of age, there were no apparent differences between MR16–1 treatment group and control group mice (Fig. 9a). Quantitative structural analyses of proximal tibia are shown in Fig. 9b. No significant differences were seen in BV, TV, BV/TV or trabecular thickness between the two groups. MR16–1 treatment therefore does not correlate with an increase in bone volume in McH/lpr-RA1 mice.

## Discussion

Previous studies have reported several murine models that develop spontaneous ankylosis [34]. For example, DBA/1 mice spontaneously develop ankylosing arthropathy in the ankle joints [35–37]. However, these mice mainly exhibit fibrous proliferation in enthesis and fibrous ankylosis, without erosion and bone destruction [34]. Furthermore, other papers reported that the intraperitoneal injection of β-1,3-glucan (curdlan) induced inflammatory arthritis and ankylosing spondylitis in SKG mice. These mice indicated dactylitis, enthesitis, synovitis and destructive arthritis [38–40]. SKG mice with the peritoneal injection of curdlan show almost full symptoms of spondyloarthritis. Unlike SKG mice, McH/lpr-RA1 mice spontaneously show synovitis, pannus formation and entheseal ossification without some specific pathogens [12]. We consider that McH/lpr-RA1 mice are a suitable experimental disease model of peripheral arthritis in spondyloarthritis. The present study explores the effects of treatment with the anti-IL-6 receptor monoclonal antibody in McH/lpr-RA1 mice. In the MR16–1 treated group, results of histomorphometric and IVIS analyses showed that the proliferation of synovium and joint destruction were suppressed. However, histological investigations confirmed that entheseal ossification and ankylosis progressed even with IL-6 blockade treatment. We propose that the anti-IL-6 receptor antibody contributes to the suppression of synovial proliferation and bone destruction in McH/lpr-RA1 mice, but that further factors are involved in the prevention of entheseal inflammation and ossification.

Changes in the bone parameters of MR16–1 treated and control mice were evaluated by micro-CT imaging. In this study there were no significant differences in the bone parameters between two groups. Published studies reported the treatment effects of MR16–1 to include increased bone volume and bone strength in DBA/1 mice with collagen-induced arthritis [22, 41]. However, in this study McH/lpr-RA1 mice did not show increased bone volume following MR16–1 treatment. In the published study, the dose of MR16–1 was much higher — 8 mg was administered to DBA/1 mice. Our different findings of the effect of MR16–1 treatment on bone volume were likely due to the different dosage of MR16–1.

SAA is related with the pathogenesis of rheumatoid arthritis, and previous reports indicated that SAA was linked to disease activity and treatment response of rheumatoid arthritis [42, 43]. SAA is a suitable acute phase reactant of mice [44, 45], and previous studies showed that MR16–1 treatment suppressed the expression level serum SAA [21, 46]. SAA can be a relevant inflammation marker and useful for predicting the treatment response in the arthritis and enthesitis in McH/lpr-RA1 mice. SAA was significantly lower in the MR16–1 treated group, and we consider that the dose of MR16–1 that was administered is sufficient to suppress inflammation in McH/lpr-RA1 mice. MR16–1 is an anti-IL-6 receptor antibody for the treatment, so it is unsurprising that there were no changes in serum IL-6 level. The results of quantitative RT-PCR showed that gene expression levels of *tnf* and *IL17* were suppressed by IL-6 signal blockade. Previous studies reported that IL-6 signal blockade by MR16–1 suppresses IL-17 signaling [47, 48]. Suppression of IL-6, TNF- ɑ and IL-17 signals may contribute to the prevention of synovitis and bone destruction in McH/lpr-RA1 mice. However, only partial prevention of entheseal ossification and joint ankylosis were seen in the MR16–1 treated group — at week 20 the histological images showed the progression of histological findings of entheseal ossification and ankylosis, regardless of the prevention of synovial proliferation and bone destruction. In the results of previous clinical trials, administration of anti-IL-6 receptor antibody was insufficient for complete treatment of ankylosing spondylitis [14]. Some studies have reported that entheseal ossification in spondyloarthritis is related to IL-17 signaling [49]. The blockade of IL-17 signaling was achieved in this study; however, this signal suppression might be insufficient, as other signaling pathways (such as IL-12, IL-22 and IL-23) could be involved in the mechanisms of entheseal ossification and ankylosis. Further studies should be performed to assess the effects of IL-12, IL-22 and IL-23 signaling blockade for treatment of entheseal ossification and ankylosis with McH/lpr-RA1 mice. We consider that McH/lpr-RA1 is a useful animal model of peripheral spondyloarthritis, and a promising tool for the evaluation and development of new treatment reagents and drug repositioning treatment in destructive arthritis and entheseal inflammation. SKG mice with the peritoneal injection of curdlan seem to be a suitable animal model of spondyloarthritis, nevertheless the evaluation and development of new treatment reagents should be assessed in different strains of mice models, including McH/lpr-RA1 mice.

The results of IVIS analyses showed that there was a significant difference between the two groups in week 14 only. These results are inconsistent with the results of histological analyses, PCR and ELISA of SAA, which indicated significant differences after week 14. We consider that the proliferation of dysfunctional blood vessels in inflamed joints of McH/lpr-RA1 mice decreases around week 18. The histological findings showed significant differences and advanced deformity in late phase arthritis, including bone erosion, pannus formation and ankylosis. The advancement of destructive arthritis and ankylosis after the decreased formation of dysfunctional blood vessels may induce the inconsistent results of IVIS and histological synovitis score.

## Conclusion

In the present study, we have demonstrated for the first time that IL-6 signal blockade with MR16–1 significantly reduces the development of synovitis and joint destruction in the murine experimental model of spontaneous arthritis and enthesitis, McH/lpr-RA1. Our results indicate that the progression of deformity associated with entheseal ossification continue even with anti-IL-6 receptor antibody treatment; indicating that further factors might be involved in the progression of entheseal ossification. McH/lpr-RA1 is a promising animal model for the elucidation of the mechanism of destructive arthritis and enthesitis, and development of new treatment.

## Data Availability

The datasets generated and/or analyzed during the current study are available from the corresponding author on reasonable request.

## Abbreviations

BV: Bone volume
cDNA: complementary DNA
CT: Computed tomography
ELISA: Enzyme-linked immunosorbent assay
ICG: Indocyanine green
IL: Interleukin
IVIS: In vivo bioluminescence imaging system
PBS: Phosphate-buffered saline
RT-PCR: Realtime polymerase chain reaction
SAA: Serum amyloid A
TNF: Tumor necrosis factor
TV: Total volume
